# Early Clinical Histotripsy Treatment of Renal Cell Carcinoma

**DOI:** 10.1007/s00270-025-04023-9

**Published:** 2025-04-28

**Authors:** Tze Min Wah, Omar Abdel-Hadi, Jacqueline Brandon, Balbir Bhambra, Hamish Glencross, Jerome Occidental, John Martin Stones, Naveen Vasudev, Selina Bhattarai, Jon Cartledge

**Affiliations:** 1 Diagnostic and Interventional Radiology, Leeds Teaching Hospitals Trust, Beckett Street, Leeds, UK; 2 Leeds Institute of Medical Research, University of Leeds, Leeds, West Yorkshire UK; 3 Department of Medical Oncology, Leeds Teaching Hospitals Trust, Beckett Street, Leeds, UK; 4 Department of Pathology, Leeds Teaching Hospitals Trust, Beckett Street, Leeds, UK; 5 Department of Urology, Leeds Teaching Hospitals Trust, Beckett Street, Leeds, UK

The incidence of renal cell carcinoma (RCC) is rising with > 13,000 new cases annually in the UK [1]. Histotripsy, a non-invasive, non-thermal, non-ionizing focal therapy, holds promise for renal tumour treatment. In March 2023, the CAIN feasibility trial (ClinicalTrials.gov, NCT05432232) using the HistoSonics System for treatment of primary solid renal tumours with histotripsy was launched [2]. Herein, we describe the first patient successfully treated with 12-month follow-up.

**Fig. 1:**
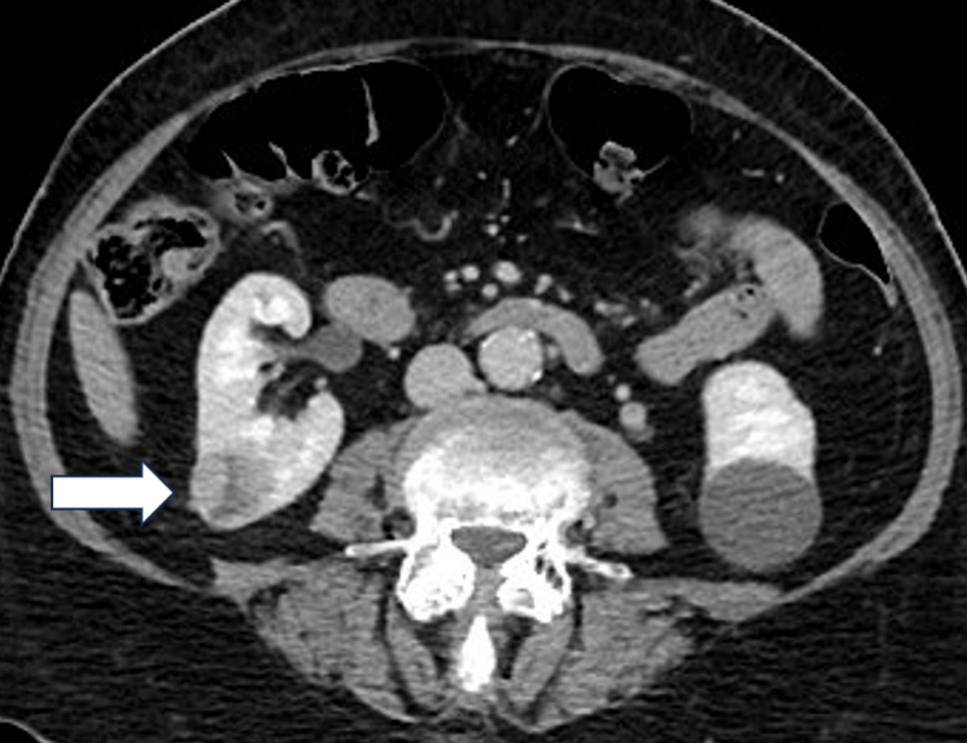
Axial contrast-enhanced CT showed an enhancing right lower pole RCC measuring 2.81 cm (maximum diameter, core lab assessed)

An 80-year-old female in a lung cancer screening trial had incidental detection of a 2.81 cm tumour in the right lower pole which had histology confirmation as grade 1 conventional clear cell renal cell carcinoma (T1aN0M0) [3]. The tumour was classified as low complexity based on the R.E.N.A.L. nephrometry score. The renal cancer multi-disciplinary team offered all treatment options: active surveillance, partial/radical nephrectomy, or image-guided ablation. The patient opted for ablation and was screened for CAIN trial participation. Following consent, CT imaging was performed (Fig. 1) to assess trial eligibility, including confirmation the tumour was ≤ 3 cm in longest diameter and did not overlap the renal pelvis, main renal vessel, ureter or other vital structure.

**Fig. 2:**
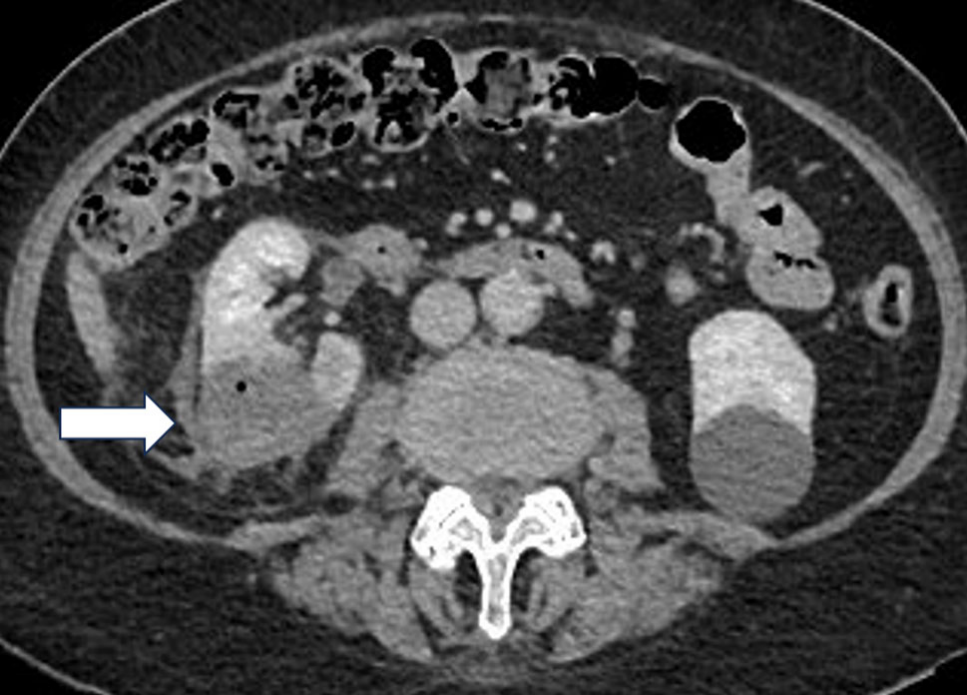
Axial contrast-enhanced CT at 36-h post-histotripsy treatment showed good treatment effect 4.27 cm (maximum diameter, core lab assessed) and completely covered the tumour. Volume outside of the planned treatment volume is due to breathing motion

**Fig. 3:**
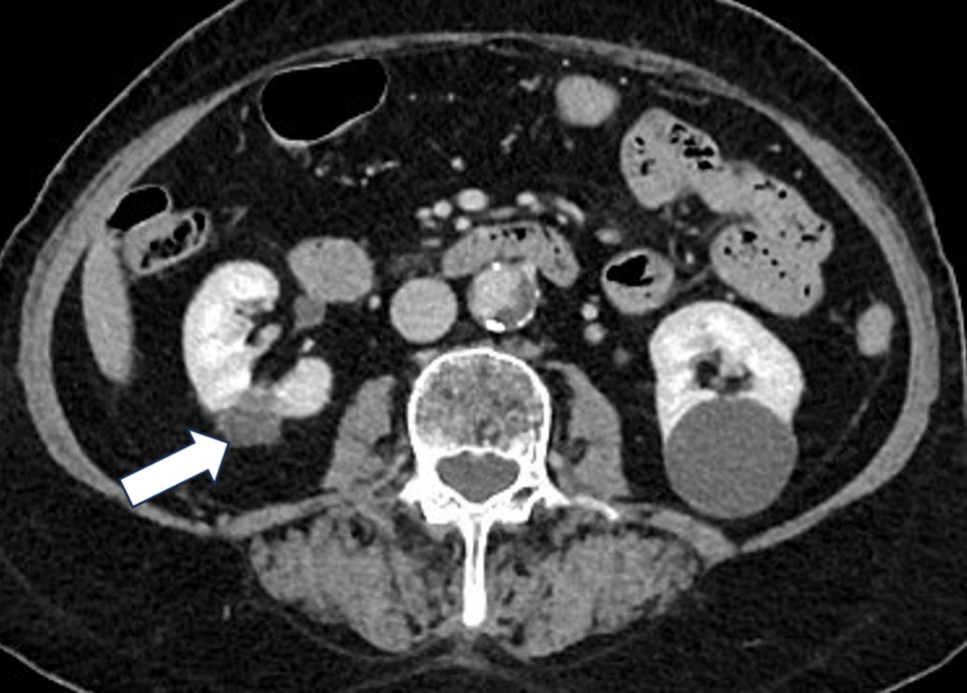
Axial contrast-enhanced CT at 1-year post-histotripsy treatment showed no evidence of residual or recurrent disease within the zone of ablation and rapidly involuting measuring 2 cm

For treatment, the patient was placed in lateral position on the surgical theatre table; her right flank was opened up by breaking the table to ensure adequate tumour visibility and shortest access route for histotripsy delivery. Ultrasound imaging was performed to determine the optimal window for the treatment head positioning. A planned treatment volume (PTV) was created using the HistoSonics System that encompassed the tumour with margins to address breathing motion and potential microscopic extension. Once treatment was initiated, the HistoSonics System continuously moved the treatment head to deliver automated therapeutic ultrasound pulses to cover the planned PTV. Throughout the procedure, the anaesthetist optimized tidal volume and positive end expiratory pressure to minimize breathing motion to maintain the tumour within the PTV. Histotripsy treatment time was 83 min. The patient was monitored post-procedure in the recovery ward for 2 h, transferred to the regular ward, and discharged home the next day. No complications were reported immediately post-procedure. Procedure-related complications of haematuria (one day post-procedure), lower abdominal pain and vomiting (both two days post-procedure) were reported; all were classified as minor (Grade I) and resolved without sequalae. Post-procedure CT at 36-h follow-up (Fig. 2) showed the histotripsy treatment zone completely covered the tumour with a maximum diameter of 4.27 cm. There was no evidence of residual or recurrent tumour at 30-, 90-, 180-day and 1-year (Fig. 3) follow-up with the zone of ablation rapidly involuting measuring 3.28, 2.82, 2.47, and 2 cm, respectively. The patient exited the trial at 168 days with no reported Clavien-Dindo Grade ≥ 3 complications.

When considering histotripsy, a renal cancer multi-disciplinary team is crucial to ensure appropriate case selection. Pre-procedural ultrasound assessment and planning is beneficial to understand treatment approach and trajectory, tumour depth in the treatment position, and assess whether histotripsy treatment is viable. During histotripsy treatment of renal tumours, the patient is often in a lateral decubitus position with the coupling water bath placed flat on the patient’s lateral chest/abdomen. The weight of the water bath must be supported with gel pad/equivalent material to avoid pressure point on the skin. The treatment head should be positioned as vertical as possible in relation to the target tumour, minimizing acute angulation of the treatment head. Additionally, respiratory motion management is vital to ensure the tumour remains within the targeted volume throughout the respiratory cycle.

This first ever global histotripsy treatment of renal cancer with follow-up out to 1 year has shown safety and durable treatment efficacy. Patient selection, pre-procedural planning, intra-procedural patient positioning and motion management are vital components to consider when embarking on this novel treatment in renal cancer.
